# Poly[aqua­(μ-pyrazine-2-carboxyl­ato-κ^3^ *N*,*O*:*O*)(μ-pyrazine-2-carboxyl­ato-κ^3^ *N*,*O*:*O*′)lead(II)]

**DOI:** 10.1107/S1600536810013188

**Published:** 2010-04-17

**Authors:** Wojciech Starosta, Janusz Leciejewicz

**Affiliations:** a Institute of Nuclear Chemistry and Technology, ul. Dorodna 16, 03-195 Warszawa, Poland

## Abstract

The polymeric structure of the title compound, [Pb(C_5_H_3_N_2_O_2_)_2_(H_2_O)]_*n*_, is built up from centrosymmetric [Pb(C_5_H_3_N_2_O_2_)_2_(H_2_O)]_2_ dimers, which are bridged by ligand carboxyl­ate O atoms. The Pb^II^ ion adopts an irregular PbN_2_O_5_ coordination polyhedron; it is chelated by one *N*,*O*-bidentate ligand and also bonds to a water O atom. A second *N*,*O*-bidentate ligand forms the dimer bridge and another bridging O atom from a nearby dimer also bonds to the Pb^II^ ion, leading to layers propagating in (100). A network of O—H⋯O hydrogen bonds operates between water O atoms (donors) and carboxyl­ate O atoms (acceptors).

## Related literature

For the crystal structures of divalent metal ions with pyrazine-2-carboxyl­ate and water ligands, see, for example: Alcock *et al.* (1996 ▶); Ptasiewicz-Bąk *et al.* (1995 ▶, 1998 ▶). The structures of lead(II) complexes with pyrazine-4-carboxyl­ate (Starosta & Leciejewicz, 2009 ▶) and pyrazine-3-carboxyl­ate ligands (Starosta & Leciejewicz, 2010 ▶) have also been reported.
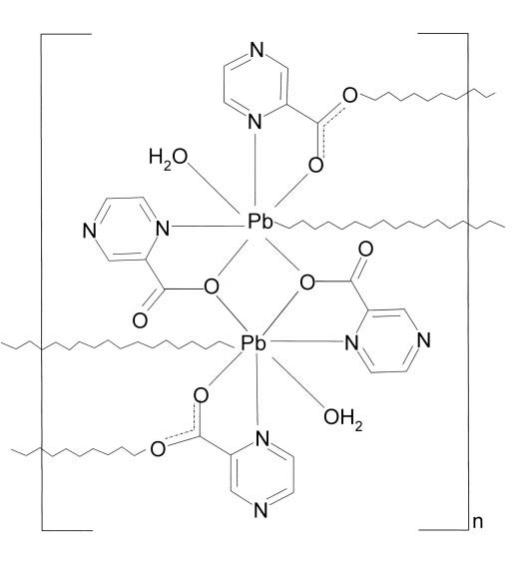


## Experimental

### 

#### Crystal data

[Pb(C_5_H_3_N_2_O_2_)_2_(H_2_O)]
*M* *_r_* = 471.39
Monoclinic, 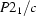

*a* = 11.098 (2) Å
*b* = 10.382 (2) Å
*c* = 11.678 (2) Å
β = 114.13 (3)°
*V* = 1228.0 (4) Å^3^
*Z* = 4
Mo *K*α radiation
μ = 13.77 mm^−1^
*T* = 293 K
0.29 × 0.16 × 0.12 mm

#### Data collection

Kuma KM-4 four-circle diffractometer
Absorption correction: analytical (*CrysAlis RED*; Oxford Diffraction, 2008 ▶) *T*_min_ = 0.135, *T*_max_ = 0.251
3579 measured reflections
3411 independent reflections
2230 reflections with *I* > 2σ(*I*)
*R*_int_ = 0.051
3 standard reflections every 200 reflections  intensity decay: 20.2%

#### Refinement

*R*[*F* ^2^ > 2σ(*F* ^2^)] = 0.058
*wR*(*F* ^2^) = 0.163
*S* = 1.02
3411 reflections
188 parameters
5 restraints
H atoms treated by a mixture of independent and constrained refinement
Δρ_max_ = 6.45 e Å^−3^
Δρ_min_ = −5.86 e Å^−3^

### 

Data collection: *KM-4 Software* (Kuma, 1996 ▶); cell refinement: *KM-4 Software*; data reduction: *DATAPROC* (Kuma, 2001 ▶); program(s) used to solve structure: *SHELXTL* (Sheldrick, 2008 ▶); program(s) used to refine structure: *SHELXTL*; molecular graphics: *SHELXTL*; software used to prepare material for publication: *SHELXTL*.

## Supplementary Material

Crystal structure: contains datablocks I, global. DOI: 10.1107/S1600536810013188/hb5383sup1.cif

Structure factors: contains datablocks I. DOI: 10.1107/S1600536810013188/hb5383Isup2.hkl

Additional supplementary materials:  crystallographic information; 3D view; checkCIF report

## Figures and Tables

**Table 1:** Selected bond lengths (Å)

Pb1—O21	2.341 (7)
Pb1—O11^i^	2.508 (7)
Pb1—O1	2.573 (9)
Pb1—O11	2.572 (8)
Pb1—N21	2.577 (9)
Pb1—N11	2.807 (9)
Pb1—O22^ii^	2.856 (8)

Symmetry codes: (i) 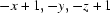; (ii) 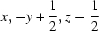.

**Table 2:** Hydrogen-bond geometry (Å, °)

*D*—H⋯*A*	*D*—H	H⋯*A*	*D*⋯*A*	*D*—H⋯*A*
O1—H2⋯O21^ii^	0.84 (2)	2.17 (5)	2.837 (13)	136 (7)
O1—H1⋯O22^iii^	0.84 (2)	2.29 (5)	2.969 (15)	139 (7)
O1—H1⋯O12^ii^	0.84 (2)	2.49 (7)	3.056 (13)	126 (7)

Symmetry codes: (ii) 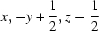; (iii) 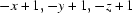.

**Fig. 1.**
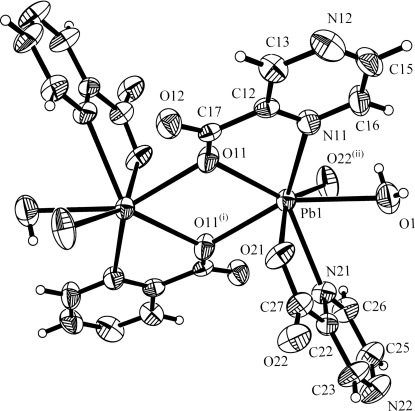
A structural unit of (1) with 50% probability displacement ellipsoids. Symmetry codes: (i) -x+1,-y,-z+1; (ii) x,-y+1/2,z-1/2.

**Fig. 2.**
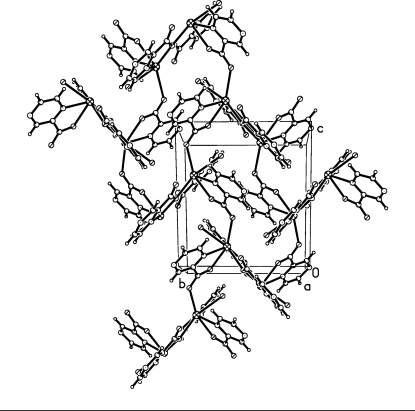
Packing diagram of the structure of (I).

## supplementary crystallographic information

### Comment

Divalent UO_2_(II) ion (Alcock *et al.*,1996), 3-d metal M(II) ions (Ptasiewicz-Bąk *et al.*,(1995), Ca(II) and Sr(II) ions (Ptasiewicz-Bąk *et al.*,1998) form with pyrazine-2-carboxylate and water ligands monomeric molecules with coordination modes characteristic for particular ions. On the other hand, the structure of a Pb(II) complex with pyridazine-4-carboxylate and water ligands is composed of dimeric molecules (Starosta & Leciejewicz, 2009), while the structure of a Pb(II) complex with pyridazine-3-carboxylate and water ligands is polymeric (Starosta & Leciejewicz, 2010). The structure of title compound (I) is composed of centrosymmetric dimeric molecules in which each of the two Pb(II) ions is cheletated by two symmetry independent ligands *via* their N,*O* bonding groups. Their planes make at the metal ion an angle of 85.1 (2)^o^ each to the other. Pb(II) ions are bridged by O11 and O11^(i)^ atoms donated by symmetry related ligands L1. The O12 and O12^(i)^ atoms do not take part in coordination. A water O atom is chelated to each metal ion. The second pair of ligand molecules L2 also coordinates the Pb(II) ions by their N,*O* bonding groups while the O22 and O22^(i)^ atoms act as bridges to Pb(II) ions in adjacent dimers. A polymeric structure is formed in this way. The coordination geometry of a Pb(II) ion is represented by a pyramid in which N11, O11, O11^(I)^ and O1 atoms form an equatorial plane [ r.m.s. 0.0083 (1) Å] with a Pb(II) ion shifted from it by 0.3079 (2) Å; N21 and O21 atoms make two apices of the pyramid while the bridging O22^(II)^ atom forms a single apex on the other side of the equatorial plane. Bond angles reveal an empty space around the metal ion between Pb—O11^(I)^and Pb—O1 bonds It may indicate the stereochemical activity of the lone 6 s^2^ electron pair of the Pb(II) ion. Pyrazine rings of both ligands are planar: r.m.s. 0.0089 (2)Å in L1 and 0.0046 (1)Å in L2. The C17/O11/O12 carboxylic group makes an angle of 6.7 (1)° with pyrazine ring L1, the carboxylic group C27/O21/O22 - an angle of 9.1 (1)° with L2. Weak hydrogen bonds operate between the coordinated water O atoms (donors) and carboxylate O21 and O22 atoms (acceptors) in adjacent dimers.

### Experimental

The title compound was synthetized by reacting boiling aqueous solution of pyrazine-2-carboxylic acid dihydrate (Aldrich) with some excess of lead(II) hydroxide. The mixture was boiled under reflux for three hours and after cooling to room temperature, filtered and left for crystallization. Few days later, colourless blocks of (I) were found after evaporation to dryness. They were extracted, washed with cold ethanol and dried in the air.

### Refinement

Water hydrogen atoms were found from Fourier maps and restrained geometrically to form hydrogen bonds. H atoms attached to pyrazine -ring C atoms were positioned geometrically and refined with a riding model. A maximum peak of 6.450 e Å^3^ (at 0.83 Å) and a deepest hole of -5.858 e Å^3^ (at 0.80 Å) were found on the final electron density map close to the Pb1 atom.

### Crystal data

**Table:**

[Pb(C_5_H_3_N_2_O_2_)_2_(H_2_O)]	*F*(000) = 872
*M**_r_* = 471.39	*D*_x_ = 2.550 Mg m^−^^3^
Monoclinic, *P*2_1_/*c*	Mo *K*α radiation, λ = 0.71073 Å
Hall symbol: -P 2ybc	Cell parameters from 25 reflections
*a* = 11.098 (2) Å	θ = 6–15°
*b* = 10.382 (2) Å	µ = 13.77 mm^−^^1^
*c* = 11.678 (2) Å	*T* = 293 K
β = 114.13 (3)°	Blocks, colourless
*V* = 1228.0 (4) Å^3^	0.29 × 0.16 × 0.12 mm
*Z* = 4	

### Data collection

**Table:**

Kuma KM-4 four-circle diffractometer	2230 reflections with *I* > 2σ(*I*)
Radiation source: fine-focus sealed tube	*R*_int_ = 0.051
graphite	θ_max_ = 30.1°, θ_min_ = 2.0°
profile data from ω/2θ scans	*h* = 0→14
Absorption correction: analytical (*CrysAlis RED*; Oxford Diffraction, 2008)	*k* = −14→0
*T*_min_ = 0.135, *T*_max_ = 0.251	*l* = −15→14
3579 measured reflections	3 standard reflections every 200 reflections
3411 independent reflections	intensity decay: 20.2%

### Refinement

**Table:**

Refinement on *F*^2^	Secondary atom site location: difference Fourier map
Least-squares matrix: full	Hydrogen site location: inferred from neighbouring sites
*R*[*F*^2^ > 2σ(*F*^2^)] = 0.058	H atoms treated by a mixture of independent and constrained refinement
*wR*(*F*^2^) = 0.163	*w* = 1/[σ^2^(*F*_o_^2^) + (0.1217*P*)^2^] where *P* = (*F*_o_^2^ + 2*F*_c_^2^)/3
*S* = 1.02	(Δ/σ)_max_ < 0.001
3411 reflections	Δρ_max_ = 6.45 e Å^−^^3^
188 parameters	Δρ_min_ = −5.86 e Å^−^^3^
5 restraints	Extinction correction: *SHELXTL* (Sheldrick, 2008), Fc^*^=kFc[1+0.001xFc^2^λ^3^/sin(2θ)]^-1/4^
Primary atom site location: structure-invariant direct methods	Extinction coefficient: 0.0154 (12)

### Special details

**Table:**

Geometry. All esds (except the esd in the dihedral angle between two l.s. planes) are estimated using the full covariance matrix. The cell esds are taken into account individually in the estimation of esds in distances, angles and torsion angles; correlations between esds in cell parameters are only used when they are defined by crystal symmetry. An approximate (isotropic) treatment of cell esds is used for estimating esds involving l.s. planes.
Refinement. Refinement of *F*^2^ against ALL reflections. The weighted *R*-factor wR and goodness of fit *S* are based on *F*^2^, conventional *R*-factors *R* are based on *F*, with *F* set to zero for negative *F*^2^. The threshold expression of *F*^2^ > σ(*F*^2^) is used only for calculating *R*-factors(gt) etc. and is not relevant to the choice of reflections for refinement. *R*-factors based on *F*^2^ are statistically about twice as large as those based on *F*, and *R*- factors based on ALL data will be even larger.

### Fractional atomic coordinates and isotropic or equivalent isotropic displacement parameters (Å^2^)

**Table:**

	*x*	*y*	*z*	*U*_iso_*/*U*_eq_	
Pb1	0.47863 (3)	0.12876 (4)	0.35712 (3)	0.02848 (19)	
O11	0.3570 (7)	−0.0031 (8)	0.4636 (8)	0.0413 (18)	
C22	0.6620 (9)	0.3784 (10)	0.5171 (10)	0.034 (2)	
O21	0.4811 (9)	0.2586 (8)	0.5220 (8)	0.0399 (18)	
N21	0.6655 (8)	0.2972 (9)	0.4344 (8)	0.0327 (18)	
C12	0.1581 (9)	0.1152 (9)	0.3625 (10)	0.0299 (19)	
C13	0.0296 (11)	0.1383 (11)	0.3473 (11)	0.039 (2)	
H13	−0.0039	0.0957	0.3980	0.047*	
C27	0.5599 (12)	0.3532 (11)	0.5717 (12)	0.040 (2)	
N22	0.8312 (12)	0.5089 (11)	0.5106 (13)	0.059 (3)	
C26	0.7546 (11)	0.3203 (12)	0.3893 (12)	0.039 (2)	
H26	0.7616	0.2642	0.3303	0.047*	
N11	0.2083 (9)	0.1695 (10)	0.2906 (9)	0.0362 (19)	
C16	0.1307 (12)	0.2491 (12)	0.2013 (12)	0.045 (3)	
H16	0.1628	0.2889	0.1481	0.054*	
C23	0.7440 (13)	0.4853 (12)	0.5559 (14)	0.050 (3)	
H23	0.7365	0.5409	0.6151	0.060*	
O1	0.4080 (11)	0.3287 (10)	0.2149 (9)	0.056 (2)	
H1	0.378 (11)	0.402 (7)	0.218 (10)	0.084*	
H2	0.385 (8)	0.312 (13)	0.138 (3)	0.084*	
O12	0.2045 (8)	−0.0170 (9)	0.5394 (8)	0.0445 (19)	
C17	0.2455 (9)	0.0251 (9)	0.4646 (9)	0.0275 (18)	
N12	−0.0479 (10)	0.2225 (11)	0.2591 (11)	0.050 (3)	
C15	0.0023 (12)	0.2730 (13)	0.1871 (12)	0.047 (3)	
H15	−0.0500	0.3278	0.1230	0.056*	
C25	0.8376 (14)	0.4253 (14)	0.4272 (15)	0.055 (3)	
H25	0.8995	0.4379	0.3935	0.066*	
O22	0.5643 (12)	0.4168 (10)	0.6610 (9)	0.054 (2)	

### Atomic displacement parameters (Å^2^)

**Table:**

	*U*^11^	*U*^22^	*U*^33^	*U*^12^	*U*^13^	*U*^23^
Pb1	0.0271 (2)	0.0296 (2)	0.0327 (3)	0.00255 (14)	0.01621 (15)	0.00445 (14)
O11	0.024 (3)	0.044 (4)	0.060 (5)	0.005 (3)	0.020 (3)	0.021 (4)
C22	0.019 (4)	0.039 (5)	0.041 (5)	−0.002 (4)	0.010 (4)	0.008 (4)
O21	0.045 (4)	0.035 (4)	0.058 (5)	−0.011 (3)	0.040 (4)	−0.012 (3)
N21	0.024 (4)	0.044 (5)	0.038 (5)	−0.005 (4)	0.021 (3)	−0.002 (4)
C12	0.018 (4)	0.035 (5)	0.038 (5)	−0.002 (3)	0.012 (3)	−0.003 (4)
C13	0.031 (5)	0.048 (7)	0.041 (6)	0.009 (4)	0.019 (4)	−0.001 (4)
C27	0.038 (6)	0.039 (6)	0.047 (6)	−0.005 (4)	0.020 (5)	−0.001 (4)
N22	0.048 (6)	0.050 (6)	0.091 (9)	−0.021 (5)	0.042 (6)	−0.007 (6)
C26	0.034 (5)	0.050 (6)	0.050 (6)	−0.004 (5)	0.032 (5)	−0.003 (5)
N11	0.027 (4)	0.043 (5)	0.040 (5)	0.001 (4)	0.016 (4)	0.009 (4)
C16	0.039 (7)	0.046 (6)	0.051 (7)	0.008 (5)	0.018 (6)	0.021 (5)
C23	0.042 (6)	0.040 (6)	0.077 (9)	−0.017 (5)	0.032 (6)	−0.012 (6)
O1	0.072 (7)	0.052 (5)	0.042 (5)	0.009 (5)	0.022 (5)	0.013 (4)
O12	0.040 (4)	0.056 (5)	0.044 (4)	0.002 (4)	0.024 (3)	0.011 (4)
C17	0.022 (4)	0.027 (4)	0.033 (5)	−0.007 (3)	0.011 (3)	−0.003 (3)
N12	0.031 (5)	0.064 (7)	0.054 (6)	0.018 (5)	0.016 (5)	−0.003 (5)
C15	0.030 (6)	0.057 (8)	0.045 (7)	0.011 (5)	0.008 (5)	0.012 (5)
C25	0.044 (7)	0.055 (8)	0.081 (10)	−0.015 (6)	0.041 (7)	0.003 (7)
O22	0.077 (6)	0.062 (6)	0.042 (5)	−0.030 (5)	0.043 (4)	−0.017 (4)

### Geometric parameters (Å, °)

**Table:**

Pb1—O21	2.341 (7)	C13—N12	1.357 (15)
Pb1—O11^i^	2.508 (7)	C13—H13	0.9300
Pb1—O1	2.573 (9)	C27—O22	1.217 (15)
Pb1—O11	2.572 (8)	N22—C23	1.302 (17)
Pb1—N21	2.577 (9)	N22—C25	1.328 (19)
Pb1—N11	2.807 (9)	C26—C25	1.378 (18)
Pb1—O22^ii^	2.856 (8)	C26—H26	0.9300
O11—C17	1.277 (12)	N11—C16	1.334 (14)
O11—Pb1^i^	2.508 (7)	C16—C15	1.388 (17)
C22—N21	1.295 (14)	C16—H16	0.9300
C22—C23	1.389 (15)	C23—H23	0.9300
C22—C27	1.532 (16)	O1—H1	0.84 (2)
O21—C27	1.285 (14)	O1—H2	0.84 (2)
N21—C26	1.318 (12)	O12—C17	1.219 (12)
C12—N11	1.310 (13)	N12—C15	1.295 (17)
C12—C13	1.385 (13)	C15—H15	0.9300
C12—C17	1.513 (13)	C25—H25	0.9300
			
O21—Pb1—O11^i^	81.6 (3)	C13—C12—C17	120.2 (9)
O21—Pb1—O1	88.0 (3)	N12—C13—C12	120.6 (11)
O11^i^—Pb1—O1	152.2 (3)	N12—C13—H13	119.7
O21—Pb1—O11	75.0 (3)	C12—C13—H13	119.7
O11^i^—Pb1—O11	70.5 (3)	O22—C27—O21	125.7 (12)
O1—Pb1—O11	131.3 (3)	O22—C27—C22	119.1 (10)
O21—Pb1—N21	65.4 (3)	O21—C27—C22	115.1 (10)
O11^i^—Pb1—N21	81.6 (3)	C23—N22—C25	116.5 (11)
O1—Pb1—N21	70.6 (3)	N21—C26—C25	121.9 (11)
O11—Pb1—N21	134.3 (3)	N21—C26—H26	119.0
O21—Pb1—N11	78.0 (3)	C25—C26—H26	119.0
O11^i^—Pb1—N11	129.8 (3)	C12—N11—C16	117.4 (10)
O1—Pb1—N11	72.0 (3)	C12—N11—Pb1	116.4 (6)
O11—Pb1—N11	60.1 (3)	C16—N11—Pb1	125.8 (8)
N21—Pb1—N11	127.6 (3)	N11—C16—C15	120.5 (11)
O21—Pb1—O22^ii^	149.2 (3)	N11—C16—H16	119.8
O11^i^—Pb1—O22^ii^	102.5 (3)	C15—C16—H16	119.8
O1—Pb1—O22^ii^	74.3 (3)	N22—C23—C22	121.0 (13)
O11—Pb1—O22^ii^	135.4 (3)	N22—C23—H23	119.5
N21—Pb1—O22^ii^	84.8 (3)	C22—C23—H23	119.5
N11—Pb1—O22^ii^	118.2 (3)	Pb1—O1—H1	137 (10)
C17—O11—Pb1^i^	119.3 (6)	Pb1—O1—H2	113 (9)
C17—O11—Pb1	125.8 (6)	H1—O1—H2	106 (3)
Pb1^i^—O11—Pb1	109.5 (3)	O12—C17—O11	124.8 (9)
N21—C22—C23	123.2 (11)	O12—C17—C12	118.7 (9)
N21—C22—C27	116.9 (9)	O11—C17—C12	116.5 (8)
C23—C22—C27	119.9 (11)	C15—N12—C13	116.5 (10)
C27—O21—Pb1	126.1 (7)	N12—C15—C16	122.9 (11)
C22—N21—C26	115.8 (10)	N12—C15—H15	118.5
C22—N21—Pb1	116.0 (6)	C16—C15—H15	118.5
C26—N21—Pb1	127.7 (8)	N22—C25—C26	121.5 (11)
N11—C12—C13	121.9 (10)	N22—C25—H25	119.2
N11—C12—C17	117.9 (8)	C26—C25—H25	119.2

Symmetry codes: (i) −*x*+1, −*y*, −*z*+1; (ii) *x*, −*y*+1/2, *z*−1/2.

### Hydrogen-bond geometry (Å, °)

**Table:**

*D*—H···*A*	*D*—H	H···*A*	*D*···*A*	*D*—H···*A*
O1—H2···O21^ii^	0.84 (2)	2.17 (5)	2.837 (13)	136 (7)
O1—H1···O22^iii^	0.84 (2)	2.29 (5)	2.969 (15)	139 (7)
O1—H1···O12^ii^	0.84 (2)	2.49 (7)	3.056 (13)	126 (7)

Symmetry codes: (ii) *x*, −*y*+1/2, *z*−1/2; (iii) −*x*+1, −*y*+1, −*z*+1.
